# Coenzyme Q10 Metabolism: A Review of Unresolved Issues

**DOI:** 10.3390/ijms24032585

**Published:** 2023-01-30

**Authors:** David Mantle, Guillermo Lopez-Lluch, Iain Parry Hargreaves

**Affiliations:** 1 Pharma Nord (UK) Ltd., Morpeth NE61 2DB, UK; 2 Department of Physiology, Anatomy and Cell Biology, Andalusian Centre of Developmental Biology, Universidad Pablo de Olavide, 41013 Seville, Spain; 3 School of Pharmacy and Biomolecular Sciences, Liverpool John Moores University, Liverpool L3 3AF, UK

**Keywords:** coenzyme Q10, bioavailability, blood-brain barrier, intracellular transporters, intravenous delivery

## Abstract

The variable success in the outcome of randomised controlled trials supplementing coenzyme Q10 (CoQ10) may in turn be associated with a number of currently unresolved issues relating to CoQ10 metabolism. In this article, we have reviewed what is currently known about these factors and where gaps in knowledge exist that need to be further elucidated. Issues addressed include (i) whether the bioavailability of CoQ10 could be improved; (ii) whether CoQ10 could be administered intravenously; (iii) whether CoQ10 could be administered via alternative routes; (iv) whether CoQ10 can cross the blood-brain barrier; (v) how CoQ10 is transported into and within target cells; (vi) why some clinical trials supplementing CoQ10 may have been unsuccessful; and (vii) which is the most appropriate tissue for the clinical assessment of CoQ10 status.

## 1. Introduction

The key role of coenzyme Q10 (CoQ10) in cell metabolism has been described in detail by numerous authors, most notably by Crane [1], from whose article the following information is summarised. Briefly, CoQ10 has a number of important functions, both within mitochondria and elsewhere within cells. Within mitochondria, CoQ10 has a key role in the generation of ATP via oxidative phosphorylation, as an electron carrier from complex I and II to complex III in the mitochondrial electron transport chain. CoQ10 is the major endogenously synthesised lipid-soluble antioxidant, protecting all types of cellular membranes from free radical-induced oxidative damage. In addition to a role in maintaining lysosomal pH, CoQ10 also has a role in the metabolism of pyrimidines, sulphides, and amino acids. CoQ10 may also mediate the expression of a number of genes, particularly those involved in inflammation.

Given the importance of CoQ10 in normal cell functioning, it is not surprising that a deficiency of CoQ10 has been implicated in a wide range of disorders [2]. Randomised controlled trials supplementing CoQ10 in such disorders have been described, with variable success in outcomes. This in turn may be associated with a number of currently unresolved factors, such as the optimal method of administration and the ability of different tissues to take up CoQ10 once absorbed from the digestive tract [3]. In this article, we have reviewed what is currently known about these factors, and where gaps in knowledge exist that need to be further elucidated.

## 2. Could the Bioavailability of CoQ10 Be Improved?

Bioavailability is defined as the proportion of an ingested substance that reaches the bloodstream following absorption from the digestive tract. Because of the particular chemical structure of CoQ10 (one of the most hydrophobic naturally occurring molecules), its bioavailability is low, typically of the order of 5% or less [3]. A number of ways of improving CoQ10 bioavailability have been described, as reviewed in this section; however, before addressing these, the mechanism of CoQ10 absorption must first be considered. What is known about the mechanism of CoQ10 has been described in detail (with a corresponding reference list) in the review by Mantle and Dybring [3], from which the following information is derived. Briefly, CoQ10 is absorbed via the same mechanism as any other lipid-soluble substance. Following ingestion and transit through the stomach, CoQ10 enters the duodenum where it is subject to the process of micellisation [3]. These spherical structures are small enough to diffuse between the intestinal villi, before breaking apart to release individual CoQ10 molecules adjacent to the surface of enterocyte cells responsible for CoQ10 absorption [3]. Variable dosage studies in humans have indicated that there is a finite capacity to absorb CoQ10 in a single dose [4], suggesting that a carrier is required to facilitate the entry of CoQ10 into enterocytes. The carrier has not been definitively identified, although the cholesterol transporter NCPC1L1 (Niemann-Pick C1 Like 1) has been suggested [5,6]. Within the enterocytes, the CoQ10 molecules are incorporated into chylomicrons. Chylomicrons are synthesised in the endoplasmic reticulum and then released from the enterocytes into the lymphatic system, from which they enter into the blood circulation. Chylomicrons in the blood carry CoQ10 to the liver, where it is then loaded primarily into LDL (low density lipoprotein) and VLDL (very low density lipoprotein) lipoprotein particles for transport around the body [3].

Of particular importance in the absorption process outlined above is the incorporation of CoQ10 into cytosolic lipid droplets, in the initial stage of chylomicron formation within the enterocytes. In general terms, cytosolic lipid droplets serve as a lipid storage pool during the post-prandial phase [7]. This retention of neutral lipids into enterocytes has been associated with the activity of liver X receptors, master regulators of cholesterol catabolism [8]. One of the functions of this process is to protect against the occurrence of hypertriglyceridemia, but this may also be responsible for the lag phase until supplemented CoQ10 is detected in the blood [9]. A number of proteins may be associated with the enterocyte lipid droplets, including CoQ10-reducing enzymes such as cytochrome b5-reductase (Cyb5R3), which in part may explain why supplemental CoQ10 reaches the blood circulation in its reduced (ubiquinol) state [10]. Further research is therefore required to develop a clearer understanding of the role of cytosolic lipid droplets in CoQ10 transit within enterocytes, since this may in turn represent a rate-controlling step in CoQ10 absorption.

The single most effective method to date for improving CoQ10 bioavailability is arguably the patented CoQ10 crystal modification process used by Pharma Nord ApS in the manufacture of their ubiquinone form CoQ10 supplements [9]. CoQ10 is produced via a yeast fermentation process in the form of polymorphic crystals, which cannot be absorbed from the digestive tract. CoQ10 can be absorbed only as individual molecules, as noted above. To be effective as a supplement, the CoQ10 crystals must therefore be dissociated first into individual CoQ10 molecules prior to absorption [9]. The above process involves changing the shape of the CoQ10 crystals in such a way as to increase the ratio of the crystals’ surface area to the volume, thus making it easier for the crystals to dissolve into single molecules at body temperature [9]. This modification to the CoQ10 crystalline form should remain in place throughout the shelf life of the CoQ10 preparation.

The value of this process was demonstrated in the clinical study by Lopez-Lluch et al. [9]. In this randomised controlled clinical trial, the bioavailability of seven CoQ10 supplements differing in formulation (CoQ10 crystal modification status, type of carrier oil, composition of other excipients, and CoQ10 oxidation state) was administered in a single 100 mg dose to the same series of 14 healthy individuals, using a crossover/washout protocol. The bioavailability of the different formulations was quantified as the area under the curve (AUC) at 48 h. The supplement that had been subject to the crystal modification process had the highest level of bioavailability, whilst the bioavailability of the same CoQ10 material that had not been subject to this process was reduced by 75%.

A second point from this study relates to the relative bioavailability of the ubiquinone and ubiquinol forms of CoQ10. The bioavailability of a ubiquinol supplemental form was approximately twice that of ubiquinone which had not been subjected to thermal crystal modification but was only 52% of ubiquinone that had been subjected to thermal crystal modification. This clearly indicates that the modification in CoQ10 crystal morphology described above is essential to improve the capacity to access enterocytes. The above finding is of relevance to claims that the ubiquinol form of supplemental CoQ10 is more bioavailable than the ubiquinone form. In addition, research carried out by the late Dr. William Judy demonstrated that under conditions simulating the environ of the stomach and small intestine in vitro, supplemental ubiquinol is largely oxidised to ubiquinone prior to entry into enterocytes [11]. Furthermore, studies supplementing ubiquinol in dogs similarly showed oxidation of the latter to ubiquinone prior to enterocyte absorption, with the subsequent conversion of ubiquinone back to ubiquinol following the passage from enterocytes into the lymphatic system [12].

A third point arising from the study by Lopez-Lluch et al. [9] is that some individuals appear to have an inherently low capacity to absorb supplemental CoQ10 into the bloodstream, even with high bioavailability formulations. The reason for this is currently unknown.

A number of studies have been carried out with the objective of improving CoQ10 bioavailability using a variety of agents; examples include polyethylene glycol [13], phosphorylated tocopherols [14], polyoxamer/polyvinyl pyrrolidine [15], and hydrolysed proteins [16]. However, again, the bioavailability of most of these formulations has not been compared directly with ubiquinone that has undergone crystal modification, the importance of which is demonstrated in the study by Lopez-Lluch et al. above [9]. In addition, none of the modified forms of CoQ10 described above have been subject to an extensive evaluation of efficacy and safety in randomised controlled trials. In comparison, the efficacy and safety of the crystal-modified form of CoQ10 have been confirmed in a number of such clinical studies, as further described in Section 6 of this article.

In summary, before claims for superior bioavailability of CoQ10 supplements based on novel formulations can be made, a comparison against the crystal-modified ubiquinone CoQ10 form should be carried out, using the same type of clinical study format as that described by Lopez-Lluch et al. [9]. In addition, outstanding issues requiring further research are: (i) to establish the identity of the carrier responsible for transporting CoQ10 molecules from the intestinal milieu into enterocytes; and (ii) to develop a clearer understanding of the role of cytosolic lipid droplets in CoQ10 transit within enterocytes, since these may, in turn, represent rate-controlling steps in CoQ10 absorption. Finally, the reason why some individuals have a low inherent capacity to absorb supplemental CoQ10 should be investigated, since the inclusion of such individuals in clinical trials could obscure trial outcomes. Given the potential limitations of the absorption of CoQ10 from the digestive tract, the question arises as to whether CoQ10 could be administered intravenously, and this is discussed in the following section.

## 3. Could CoQ10 Be Administered Intravenously?

Given the low bioavailability of CoQ10 when administered orally as outlined above, the administration of CoQ10 via intravenous injection is an obvious alternative. However, the potential problem with this approach is that there is no appreciable circulation of unbound CoQ10 in the blood; CoQ10 is transported in the blood bound principally to LDL- and VLDL-cholesterol, with a relatively small amount of CoQ10 associated with HDL cholesterol [3]. The question, therefore, arises whether it is necessary to bind CoQ10 to LDL- or VLDL-cholesterol prior to injection, or whether an alternative type of carrier or solubilisation method could be utilised.

To date, no clinical studies were identified in which CoQ10 (in any form) was administered intravenously to human subjects. A number of studies have been carried out in various animal species in which CoQ10 was administered intravenously, although no studies were identified in which CoQ10 was specifically coupled to LDL- or VLDL-cholesterol. Studies in animal models typically use micellar or liposomal formulations of CoQ10 for intravenous injection. Examples include the micellisation of CoQ10 using the surfactant caspofungin [17] to increase plasma and tissue CoQ10 levels; following intravenous injection in mice, the micellisation of CoQ10 using HCO-60 (polyoxyethylene hydrogenated castor oil-60) to increase CoQ10 levels in liver tissue following intravenous injection in guinea pigs [18]; and intravenous injection of liposomal CoQ10 to increase myocardial CoQ10 levels in rats [19]. Where these types of animal models were used to study pathological processes, intravenous injection of CoQ10 in micellar or liposomal formulations typically resulted in significant improvements in the parameters being studied. For example, in the latter study, increased levels of myocardial CoQ10 resulted in improved tolerance to subsequent ischaemic reperfusion injury.

In summary, clinical studies are required to confirm the safety of the above types of micellar or liposomal CoQ10 formulations for intravenous injection in humans together with further studies to determine the potential of CoQ10 bound to LDL- or VLDL cholesterol carriers for similar intravenous administration. In vitro studies have demonstrated that the addition of alcoholic solutions of CoQ10 to foetal bovine serum results in the incorporation of CoQ10 principally to LDL-cholesterol (Moreno Fernández-Ayala, personal communication), suggesting that perfusion of serum with CoQ10 could be a good strategy to be used in human studies.

## 4. Could CoQ10 Be Administered via Alternative Routes?

In this section of the article, we have reviewed possible alternative routes of CoQ10 administration, including intraperitoneal, intramuscular, subcutaneous, and topical routes. In general terms, the rate of absorption is greatest for intraperitoneal injection, followed by the intramuscular and subcutaneous routes. With regard to clinical studies, there are no listings in the medical literature relating to the administration of CoQ10 via intraperitoneal, intramuscular, or subcutaneous injection. However, a number of clinical studies have described the topical application of CoQ10 to the skin, the gums, or the surface of the eyes. With regard to skin, topical application of a cream containing 350 uM CoQ10 over a 2-week period resulted in a significant increase in CoQ10 levels in the outermost layer of the skin [20], where it helped improve skin elasticity and reduce photoaging and wrinkle formation [21]. Topical application (over a four-to-six-week period) of various proprietary CoQ10 formulations to the gums of patients with periodontal disease resulted in a significant improvement in plaque index, gingival index, gingival bleeding index, and probing pocket depth, compared to scaling and planing only [22,23,24]. Topical application of CoQ10 in the form of proprietary eye drops has been used to improve healing in corneal ulcers [25] and to improve visual function in glaucoma patients [26].

With regard to animal models, the greatest number of reports listed in the literature related to the administration of CoQ10 via the intraperitoneal route (typically 10–20 mg/kg body weight). This, in turn, is a reflection of the common use of this route to administer test substances in animals, particularly rats and mice, because of the rapidity of absorption. Because of the high number of such studies, we have not attempted to include all of these in the present article, but recent examples include the neuroprotective effect following intraperitoneal injection of CoQ10 in a rat model of Alzheimer’s disease [27] and the protective effect of intraperitoneally injected CoQ10 against vincristine-induced peripheral neuropathy in rats [28].

The intramuscular injection of CoQ10 has been described in several animal models. Intramuscular administration of CoQ10, administered at a dose of 20 mg/kg (as a 1% solution in Tween 80), was described by Eleawa et al. [29] to reduce the effects of myocardial damage following induced myocardial infarction in the rat. Intramuscular injection of CoQ10 (12 mg/kg) was described in a study on the effects of aortic stenosis on oxidative stress in rabbits [30]. Intramuscular injection of CoQ10 (20 mg/kg) improved tolerance to ischaemic reperfusion injury in rats [31]. Intramuscular injection of CoQ10 (emulsified with ethanol) was used in an investigation into lymphocyte energy metabolism in tumour-bearing rats [32]. Subcutaneous injection of the CoQ10 analogue MitoQ (3–5 mg/kg) provided limited protection against amikacin-induced hearing loss and cochlear damage in guinea pigs [33].

In summary, data from the above studies provide evidence for the effective action of CoQ10 when administered by intraperitoneal, intramuscular, or subcutaneous routes in the various animal models of disease. The potential beneficial action of CoQ10 resulting from these administration routes in human subjects is an area for future research.

## 5. Does CoQ10 Cross the Blood-Brain Barrier?

There is some evidence that supplementary CoQ10 can penetrate the blood–brain barrier in animal species. For example, oral administration of CoQ10 (200 mg/kg/day for 1–2 months) in 12–24-month-old rats resulted in a subsequent increase of 30–40% in cerebral cortex CoQ10 levels [34]. Similarly, oral administration of emulsified CoQ10 (150 uM for 7 days) to 15-month-old mice increased CoQ10 levels in brain mitochondria [35]. However, whether CoQ10 can cross the blood–brain barrier in man has yet to be established. In this regard, synthetic analogues of CoQ10 such as idebenone or mitoquinone have been developed with the intention of improving blood–brain barrier penetration or mitochondria-specific targeting, although the efficacy and safety of such compounds have yet to be fully established in clinical studies [36].

**Figure 1:**
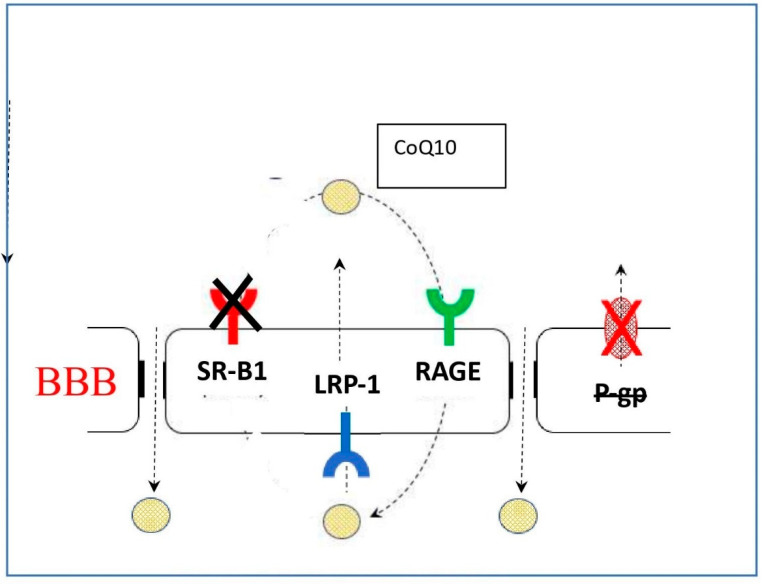
The NET direction of transport reverses with CoQ_10_ deficiency, delivering CoQ_10_ toward brain. BBB: Blood–brain barrier; SR-B1: Scavenger receptor B1; LRP-1: Low Density Lipoprotein related protein -1; RAGE: Receptor Advanced Glycation End-products; P gp: P-glycoprotein transporter.

To further elucidate the mechanism through which CoQ10 may access the blood–brain barrier, using a model system based on porcine brain endothelial cells, Wainwright et al. [37] identified lipoprotein-associated CoQ10 transcytosis in both directions across the in vitro BBB. CoQ10 uptake via SR-B1 (Scavenger Receptor) and RAGE (Receptor for Advanced Glycation Endproducts) receptors was matched by efflux via LDLR (Low Density Lipoprotein Receptor) transporters, resulting in no “net” transport across the BBB. When CoQ10 deficiency was induced in the model (using p-aminobenzoic acid), BBB tight junctions were disrupted and CoQ10 “net” transport to the brain side increased (Figure 1).

Within this area of research, a study by Park et al. [38] is of particular note. In this study, using a rat model of Parkinson’s disease, continuous intrastriatal delivery of low-dose CoQ10 (some four orders of magnitude lower than orally administered CoQ10) showed significant benefits in terms of dopaminergic neuronal loss, as well as behavioural benefits. Whether such an invasive regime could be used for the treatment of Parkinson’s patients is currently an unresolved question. To date, only two randomised controlled trials involving intrastriatal intervention (with agents other than CoQ10) in Parkinson’s disease patients have been reported, both of which involved surgical transplantation, and both of which provided no significant symptomatic benefit [39,40].

With regard to the transport of substances into the brain, a point to consider relates to a difference in dosing regimens between the Phase II and Phase III trials of CoQ10 in Parkinson’s disease. In the Phase II study, CoQ10 in daily doses of 300 mg, 600 mg, or 1200 mg was administered to Parkinson’s disease patients, resulting in a significant slowing of functional decline [41]. In the Phase III study, daily doses of CoQ10 (1200 mg or 2400 mg) were administered to Parkinson’s disease patients, together with a daily dose of 1200 IU of vitamin E, and there was no evidence of significant symptomatic benefit, in contrast to the outcome of the Phase II study [42]. As with the transport of CoQ10 into the brain, the transport of vitamin E into the brain is also poorly understood [43]. The question, therefore, arises as to whether the co-administration of a high dose of vitamin E could have inhibited access to the brain for CoQ10, via competition for shared lipoprotein or other carrier types, for example.

In summary, further work is required (i) to definitively establish whether supplemental CoQ10 is able to cross the blood–brain barrier in human subjects, (ii) and to identify whether specific transporters are involved in the latter process and whether competition with such transporters may occur with other lipid-type substances such as vitamin E.

## 6. How Is CoQ10 Transported into and within Target Cells?

An outstanding issue is the elucidation of the mechanism whereby supplemental CoQ10 is transferred from the blood into cells: Is this a diffusion-controlled process in which the level of CoQ10 in the blood needs to be greater than that in the cell to enable net access, or is this a transporter-mediated process? At present, this question is largely unresolved, although the finding that the blood CoQ10 level needs to be raised to at least 2.5 ug/mL to be of benefit in heart failure [44] suggests that a diffusion process may be operating.

Within cells, CoQ10 is located primarily (approximately 80% of total cell content) in the mitochondria but is also found in the plasma membrane, and membranes of the endoplasmic reticulum, Golgi apparatus, peroxisomes, and lysosomes. The principal site of synthesis is in the inner mitochondrial membrane [45]. Some system of transport must therefore exist to move CoQ10 from its principal site of synthesis to where it is required elsewhere within the cell. It has been demonstrated that exogenous CoQ10 moves into human cells by using an endomembrane system [46], although the regulation of the transfer from the inner mitochondrial membrane to the endomembrane system remains to be clarified.

Two putative CoQ10 transporters have been identified. In yeast, two proteins (Cqd1 and Cqd2) of the UbiB family were identified as mediating the intracellular distribution of mitochondrial-derived CoQ6 (the principal form of CoQ in yeast) [47]. UbiB family proteins are widely distributed, and there are five UbiB proteins (ADCK 1-5). It remains to be seen whether there is functional conservation between Cqd1 and Cqd2 and their putative human analogues. Another type of CoQ10 transporter of relevance to human CoQ10 metabolism was identified by Jin et al. [48] as saposin B, a lipid-binding protein. CoQ10 complexed with saposin B was identified in several types of human cells.

## 7. Why Has CoQ10 Administration Been Unsuccessful in Some Clinical Trials?

A number of factors may contribute to the unsuccessful outcome of randomised controlled trials supplementing CoQ10. Some of these can be addressed by reference to a randomised controlled trial in which CoQ10 supplementation was eminently successful. The randomised controlled trial in question is the Q-SYMBIO study led by the Danish cardiologist Dr. *Svend* Aage *Mortensen* of Rigshospitalet in Copenhagen [49]. In this study, over a two-year period, 420 patients with moderate to severe heart failure were randomised to receive 100 mg of CoQ10 three times daily or placebo, in addition to standard therapy. Supplementation with CoQ10 significantly reduced the relative risk of both cardiac-related deaths (43%) and all-cause mortality (42%). There was no significant difference in adverse events between the CoQ10-treated and placebo groups over the duration of the study.

Firstly, the investigators used the crystal-modified form of supplemental CoQ10, the bioavailability of which had been previously documented in human subjects [50]. Secondly, the blood levels of CoQ10 were measured following supplementation, to confirm that the dosage of CoQ10 employed was of sufficient magnitude and duration to raise the circulatory CoQ10 level sufficiently to facilitate the transfer of CoQ10 into cardiac tissue [51]. Thirdly, in terms of the number of participants, the trial was adequately powered to address the major clinical endpoints.

In randomised controlled trials in which CoQ10 supplementation is reportedly unsuccessful, one might question (i) whether the bioavailability of the CoQ10 supplement used had been previously confirmed in human subjects; (ii) and whether the circulatory level of CoQ10 was measured after supplementation, to confirm that the dosage employed, was sufficiently absorbed. In the latter case, such measurements are not always carried out. For example, in a randomised controlled trial supplementing CoQ10 in heart failure patients with preserved ejection fraction, no significant benefit on heart function was reported [52]; however, blood levels of CoQ10 post-supplementation do not appear to have been measured, and it is only assumed that the supplemental CoQ10 was sufficiently absorbed. In addition, the CoQ10 supplement utilised in this study had not been subject to the crystal modification process described above, and the number of trial participants (39) was relatively small.

In the case of unsuccessful randomised controlled trials supplementing CoQ10 in neurodegenerative disorders, a further complicating factor is whether supplementary CoQ10 is able to cross the blood–brain barrier; as noted in Section 4 of this article, this has not been definitively established in human subjects. It is of note that oral administration of CoQ10 (100 mg/day for 2 weeks, then 300 mg/day for 2 weeks) in five healthy subjects resulted in a significant increase in plasma CoQ10 levels but no change in CSF CoQ10 levels [53]. These data suggest that, in this study, either CoQ10 was not able to access the CNS from blood, or that CoQ10 was being removed from the CNS, for example by the P-glycoprotein transporter [54].

A final point to consider Is whether supplementation with CoQ10 alone is sufficient to resolve mitochondrial dysfunction, where this has been implicated in the pathogenesis of disorders such as Parkinson’s disease. In addition to CoQ10, a number of other nutrient-type substances are known to be of importance for normal mitochondrial function. These include the B vitamins B1, B2, and B3 (a precursor of NADH), which have roles in the tricarboxylic acid (TCA) cycle, selenium and vitamin D3 with roles in oxidative phosphorylation, and L-carnitine which is responsible for the transport of fatty acids into mitochondria prior to utilisation in the TCA cycle. Depleted levels of CoQ10 have been reported in plasma, platelets, and cerebral cortical tissue from Parkinson’s disease patients [55,56,57]. However, a stage III clinical trial in which Parkinson’s disease patients were supplemented with CoQ10 (1200–2400 mg/day) was surprisingly found to be of no clinical benefit [42]. In addition to the deficiency of CoQ10 in Parkinson’s disease, deficiencies in selenium [58], vitamins B1, B2, and B3 [59,60,61], L-carnitine [62], and vitamin D3 [63] have also been implicated in the pathogenesis of this disorder. One might therefore speculate on the outcome of a clinical study on Parkinson’s disease in which patients were supplemented with a combination of CoQ10, selenium, vitamins B1-B3, L-carnitine, and vitamin D3 in appropriate dosages, bearing in mind the possible competition of these supplements for access to cellular transporters, as outlined in Section 5 of this article.

## 8. Which Is the Most Appropriate Tissue to Use for the Clinical Assessment of CoQ10 Status?

Clinical assessment of CoQ10 status is generally based on the measurement of CoQ10 in plasma. However, concerns have been raised as to whether the level of CoQ10 in plasma is representative of the levels in other tissues. This is in part because plasma CoQ10 status is influenced by both dietary supply and liver biosynthesis [64]. This is in contrast to other tissues, which are dependent upon de novo biosynthesis to maintain their CoQ10 status [65]. In addition, plasma CoQ10 status is also dependent upon the concentration of the lipoproteins that are the major carriers of CoQ10 in circulation, with approximately 58% of total plasma CoQ10 being associated with the LDL fraction [66,67]. In this regard, it has been suggested that plasma CoQ10 levels should be expressed as a ratio relative to either total plasma cholesterol or LDL cholesterol, in order to take into account the level of circulatory lipoproteins [68,69]. However, in view of its dependence upon both dietary intake and lipoprotein concentration, plasma CoQ10 status may not truly reflect cellular levels [70]. In contrast to plasma, skeletal muscle is considered the most appropriate tissue for CoQ10 assessment, and this tissue has been used to diagnose CoQ10 deficiency since the first cases of CoQ10 deficiency were reported by Ogasahara and colleagues in 1989 [71]. However, obtaining a muscle biopsy is an invasive procedure, and it may not be appropriate to obtain a muscle biopsy from all patients with a suspected CoQ10 deficiency or to use this parameter to monitor tissue CoQ10 status following supplementation. Assessment of blood mononuclear cells has been suggested as an alternative surrogate to evaluate endogenous CoQ10 status [70]. Mononuclear cells are easily isolated from EDTA/Li-heparin blood samples, and the CoQ10 status of these cells has been reported to correlate with that of skeletal muscle [70]. Blood mononuclear cells are also reported to reflect changes in cellular status following supplementation [72]. Platelets have also been utilised as surrogates to evaluate endogenous CoQ10 levels in clinical studies [73,74]. Furthermore, the CoQ10 status of platelets was also found to increase following CoQ10 supplementation, indicating that these cell fragments may also be used to monitor the effects of CoQ10 supplementation on endogenous levels [75]. However, it has been suggested that there may be tissue-specific isoenzymes present within the CoQ10 biosynthetic pathway and, therefore, the CoQ10 status of one tissue may not reflect that of another [71]. The measurement of CoQ10 in skin fibroblasts, CSF, and urine has also been described. The use of skin fibroblasts has been reported to give false negative results in some cases of patients with muscle CoQ10 deficiency [76]. Measurement of CoQ10 in urine has been suggested as a surrogate marker of renal CoQ10 status, although this has yet to be demonstrated.

In summary, a recent study by Paredes-Fuentes et al. [77] is of relevance to the above discussion. In this study, CoQ10 levels were determined in a range of tissues (plasma, blood mononuclear cells (BMCs), platelets, urinary cells, and skeletal muscle) from a group of 11 healthy individuals before and after CoQ10 supplementation. The CoQ10 content in the different samples was analyzed by HPLC coupled with electrochemical detection. Whilst plasma CoQ10 levels (expressed either in absolute terms or relative to total cholesterol) significantly increased following a 1-month supplementation regime, there was no corresponding significant increase in CoQ10 levels in any of the other tissues measured. This study, therefore, supports the view that plasma CoQ10 levels are not representative of CoQ10 levels in other tissues, although, as indicated in Table 1, it generally used to assess circulatory level of CoQ10 following supplementation.

## 9. Conclusions

From the information presented above, it will be apparent that there are still a considerable number of issues to be resolved relating to CoQ10 metabolism. Firstly, a more detailed understanding of the mechanism by which CoQ10 molecules are transported from the intestinal milieu into enterocytes (particularly the identity of putative CoQ10 carriers), and subsequently via the lymphatic system into the bloodstream, needs to be developed. This, in turn, may provide a basis for improving the initial bioavailability of supplemental CoQ10, as well as a rationale as to why some individuals have a low inherent capacity to absorb supplemental CoQ10. Secondly, the mechanism whereby supplemental CoQ10 is transferred from the blood into target cells needs to be elucidated, i.e., whether this is a diffusion-controlled process in which the level of CoQ10 in the blood needs to be greater than that in the cell to enable net access, or whether this a transporter-mediated process. In particular, whether exogenous CoQ10 is able to cross the blood–brain barrier in humans needs to be established, since this may explain some of the disappointing outcomes of clinical trials supplementing CoQ10 in various neurological disorders. Thirdly, in the event that the oral bioavailability of CoQ10 cannot be improved, or that CoQ10 cannot cross the blood–brain barrier in humans, alternative routes of administration (showing promise in experimental models) should be further developed for clinical application; these include intravenous, intraperitoneal, and intramuscular routes (intrastriatal administration might also be justified in some neurological disorders). Finally, although the measurement of CoQ10 levels in plasma is widely used to identify CoQ10 deficiencies or to monitor CoQ10 levels following supplementation, further work needs to be undertaken to establish how representative plasma CoQ10 levels are of the levels of CoQ10 in other tissues.

## Figures and Tables

**Table 1:** Major pre-clinical and clinical trials referenced in the text.

Study/Ref	Condition	Dose/Duration	Outcome
Lopez-Lluch [9]	Double-blind crossover bioavailability over a 48 h period in young healthy study participants, aged 18–33 years	Single-dose 100 mg of various CoQ10 formulations	A ubiquinone formulation in a soy carrier oil and subject to a patented thermal crystal dissolution process gave significantly better bioavailability than a ubiquinol formulation and the same ubiquinone formulation without exposure to the thermal crystal dissolution process.
Wainwright [35]	Extent and mechanism of CoQ10 access through the blood-brain barrier	No information provided.	Identified lipoprotein-associated CoQ10 transcytosis in both directions across the in vitro BBB. Demonstrated CoQ10 net transport to the brain in conditions of CoQ10 deficiency.
Park [36]	Rat study	Low-dose = four orders of magnitude lower than orally administered CoQ10 dosages	Continuous intrastriatal delivery of low-dose CoQ10 showed significant benefits in terms of dopaminergic neuronal loss as well as behavioural benefits.
Shults [39]	Parkinson’s disease patients	CoQ10 in daily doses of 300 mg, 600 mg, or 1200 mg	CoQ10 supplementation resulted in a significant slowing of functional decline.
Beal [40]	Parkinson’s disease patients	CoQ10 in daily doses of 1200 mg or 2400 mg	Showed no evidence of significant symptomatic benefit.
Fernández-Ayala [43]	Cell culture study	No information provided.	Demonstrated that exogenous CoQ10 moves into human cells by using an endomembrane system.
Mortensen [46]	RCT of chronic heart failure patients	CoQ10 3 × 100 mg daily at separate meals	Demonstrated significant improvement of symptoms and survival. Significantly reduced both cardiovascular mortality and all-cause mortality.
Weis [47]	Randomized crossover trial in healthy volunteers, aged 24–30 years	Single-dose 100 mg of various CoQ10 formulations	A ubiquinone formulation in a soy carrier oil and subject to a patented thermal crystal dissolution process gave significantly better bioavailability than the other test formulations.

## Data Availability

Not applicable.
